# Optimizing Electronic Medical Records for an Acute Decompensated Heart Failure Registry: A Mixed-Methods Analysis of Data Completeness and Coding Practices at a National Cardiovascular Center in Indonesia

**DOI:** 10.7759/cureus.100876

**Published:** 2026-01-05

**Authors:** Richard Bun, Wiku B Adisasmito, Bambang B Siswanto

**Affiliations:** 1 Faculty of Public Health, Universitas Indonesia, Depok, IDN; 2 Department of Cardiology and Vascular Medicine, Universitas Indonesia, Jakarta, IDN

**Keywords:** acute decompensated heart failure (adhf), clinical registry, electronic medical records (emr), indonesia, southeast asian

## Abstract

Introduction

Clinical registries are essential for monitoring heart failure (HF) care quality, yet their effectiveness depends on the availability of structured data within electronic medical records (EMRs). This study aimed to analyze the completeness of core data elements and identify systemic barriers to documentation for patients with acute decompensated heart failure (ADHF) at the National Cardiovascular Center Harapan Kita in Jakarta, Indonesia.

Materials and methods

A mixed-methods sequential explanatory design was employed. Phase one involved a retrospective quantitative analysis of 305 EMRs of patients with ADHF admitted between January 2024 and January 2025. Data were extracted across 82 core variables harmonized with American College of Cardiology/American Heart Association (ACC/AHA) and European Society of Cardiology (ESC) EuroHeart standards. Phase two consisted of in-depth interviews with eight key informants, including cardiologists, residents, nurses, medical records staff and the head of the hospital information system, to explore the root causes of documentation gaps.

Results

The aggregate data completeness was 77.2%. However, disparities existed between the domains. Documentation for inpatient therapy was near-universal (99.5%), whereas medical history documentation was low (40.4%). Critical registry variables such as New York Heart Association (NYHA) functional class, discharge weight, and National Identity Number (NIK) had 0% structured completeness. However, qualitative findings revealed these parameters were frequently present in unstructured free-text narratives, indicating a data capture gap rather than a clinical one. This was driven by a preference for Stevenson profiles (wet/dry) in acute settings and terminological mismatches. The analysis confirmed that documentation habits are primarily shaped by reimbursement incentives and technical EMR limitations rather than clinical oversight.

Conclusions

While the institution possesses a strong foundation for administrative data, the current EMR usage prioritizes billing over clinical relevance. Developing a functional, impactful clinical registry requires transitioning from free-text narratives to structured data entry, implementing mandatory fields for high-value clinical and prognostic markers, and automating data synchronization between EMR modules. Establishing this high-quality data infrastructure is critical for future applications in predictive analytics or even artificial intelligence, enabling precision medicine approaches tailored specifically to the unique HF demographic in Southeast Asia, including Indonesia.

## Introduction

Cardiovascular disease remains the leading cause of mortality globally, accounting for approximately 32% of all deaths [1]. In Indonesia, heart failure (HF) represents a significant public health challenge, with the 2018 Basic Health Research (Riskesdas) estimating the prevalence of heart disease at 1.5% [2]. The economic burden is heavy; in 2023 alone, the Indonesian National Health Insurance (BPJS Kesehatan) expended over IDR 23.5 trillion on cardiovascular care, a figure driven largely by high readmission rates associated with acute decompensated heart failure (ADHF) [3].

To address this burden, the implementation of clinical cardiovascular registries is crucial. Registries serve as the foundation for a "Learning Health System," enabling hospitals to benchmark outcomes against international standards and identify gaps in guideline-directed medical therapy (GDMT) [4]. Previous regional initiatives, such as the ASIAN-HF registry, have highlighted that Asian HF patients present with a unique phenotype - younger, with a higher burden of non-cardiac comorbidities compared to Western populations [5,6]. Capturing these local, unique characteristics requires robust, granular data.

Currently, the Indonesian Ministry of Health is accelerating digital transformation through the "SatuSehat" platform, aiming to integrate patient data across the archipelago. The utility of this digital infrastructure depends entirely on the quality of data entry at the point of care. A major challenge in hospital systems, particularly in lower- and middle-income countries (LMICs), is the low utilization of data and failing to convert data into necessary clinical information. While administrative reporting data is usually satisfactory, clinical variables essential for risk stratification often remain hidden in unstructured narrative text [7]. Furthermore, data reliability remains a barrier to effective registry implementation.

A systematic review of national cardiac registries by Dawson et al. noted that validation processes are often inadequate, and administrative datasets are particularly clinically inaccurate because coding is frequently performed by administrators rather than clinical staff. This phenomenon is, to an extent, natural, considering the early implementation of registries is often for billing-driven purposes [7]. Consequently, diagnostic codes may be selected to align with reimbursement structures rather than clinical precision. This creates a risk that the digital record may fail to capture specific phenotypes (e.g., distinguishing HF with reduced ejection fraction (HFrEF) from HF with preserved ejection fraction (HFpEF)), thereby compromising the registry's utility for research [8,9].

This study was conducted at the National Cardiovascular Center Harapan Kita (RSJPDHK), the national referral center for cardiovascular care in Indonesia. The study aims to (1) quantify the completeness of core data elements required for an acute decompensated HF registry based on international standards, and (2) utilize qualitative inquiry to explain the systemic and technical barriers to high-quality clinical documentation. Specifically, this study seeks to distinguish between the absence of clinical care and the absence of structured data, while evaluating how reimbursement-driven workflows might influence the availability of granular research data.

## Materials and methods

Study design and setting

This study utilized a mixed-methods sequential explanatory design. The study was conducted at the National Cardiovascular Center Harapan Kita, Jakarta, Indonesia. Ethical approval was obtained from the Institutional Review Board (approval no. DP.04.03/KEP197/EC121/2025, Protocol No. 251124131). The study combined a retrospective quantitative analysis of medical records with qualitative semi-structured interviews to explain the statistical findings.

Quantitative Phase

A retrospective audit was performed on the electronic medical records (EMRs) of patients discharged between January 2024 and January 2025. The inclusion criteria were adult patients (≥18 years) with a primary diagnosis of ADHF. While the initial protocol sought specific International Statistical Classification of Diseases and Related Health Problems, Tenth Revision (ICD-10) codes (I50.21, I50.31), preliminary queries revealed these were never used; thus, the inclusion was expanded to patients with a clinical diagnosis of ADHF, with the records being coded as I50.9 (Heart failure, unspecified).

The sample size was calculated using the Lemeshow formula for a single population proportion [10]. Based on an estimated population of approximately 1,600 annual ADHF admissions, a 95% confidence level, and a 5% margin of error, the required sample size was determined to be 305 records. Samples were selected using systematic random sampling.

Data Instrument and Variables

We utilized a data dictionary comprising 82 distinct variables harmonized with the 2021 American College of Cardiology/American Heart Association (ACC/AHA) Key Data Elements and the European Society of Cardiology (ESC) EuroHeart HF data standards [9,11]. These variables were agreed by the researchers and categorized into seven domains, summarized in Table 1.

**Table 1:** Analysis domains and key variables CKD: Chronic Kidney Disease; ECG: Electrocardiogram; GDMT: Guideline-Directed Medical Therapy; IV: Intravenous; LVEF: Left Ventricular Ejection Fraction; NIK: Nomor Induk Kependudukan (National Identity Number); NYHA: New York Heart Association.

Domain	Number of variables	Key variables
Demographics & administrative	11	Age, sex, ethnicity, payer source, National ID (NIK)
Medical history	23	Comorbidities (e.g., hypertension, diabetes, CKD)
Clinical assessment	12	Vital signs, NYHA class, signs of congestion
Diagnostics	12	Labs (e.g., creatinine, electrolytes), ECG, Echocardiography (LVEF), chest radiography
Diagnosis & classification	4	Phenotype, etiology
Inpatient therapy	5	IV diuretics, inotropes, vasodilators
Discharge & outcomes	15	GDMT prescription, discharge status, discharge weight

A variable was considered "complete" if a valid value was present in the designated structured field or explicitly documented in the clinical summary. Variables marked "not applicable" (e.g., allergic reactions to medication) were excluded from the denominator. Completeness rates were calculated as percentages.

Qualitative Phase

To understand the "why" behind the quantitative data, we employed purposive sampling to recruit eight key informants involved in the documentation ecosystem. The panel included: three attending cardiologists (Dokter Penanggung Jawab Pelayanan or DPJP), two senior cardiology residents (Program Pendidikan Dokter Spesialis or PPDS), one head nurse, one medical records/quality assurance officer, and the head of the hospital information system. Semi-structured interviews lasting 20-30 minutes were conducted. The interview guide focused on workflow challenges, EMR usability, and the rationale behind specific coding practices. Interviews were audio-recorded, transcribed verbatim, and analyzed using thematic analysis to identify recurring barriers.

Data Analysis

Descriptive statistics (frequencies and percentages) were calculated for the quantitative data using Microsoft Excel (Microsoft Corp., Redmond, WA, USA). For the qualitative phase, transcripts were coded independently by two researchers and categorized to identify recurring themes regarding documentation barriers. Emerging themes were cross-referenced, and discrepancies in coding were resolved through consensus meetings to establish inter-rater reliability. The final thematic framework was agreed upon by all investigators.

## Results

Patient demographics and characteristics

The demographic profile of the 305 patients reflected the "Asian Phenotype" of HF, characterized by a mean age of 55.98 ± 15.30 years, significantly younger than cohorts in Western registries [5]. The majority of patients were male subjects (n=204; 66.9%) and covered by the national insurance scheme (BPJS Kesehatan) (n=297; 97.4%). Clinical outcomes showed a median length of stay (LOS) of six days (Range: 1-40 days) and an in-hospital mortality rate of 10 (3.3%). Detailed baseline characteristics are presented in Table 2.

**Table 2:** Baseline characteristics of patients BPJS Kesehatan: Badan Penyelenggara Jaminan Sosial Kesehatan (Indonesia's National Health Insurance); OOP: Out-of-pocket. *Data for length of stay is presented as Median (Min-Max) due to non-normal distribution.

Characteristic	Category	Value n (%) or Mean ± SD
Demographics		
Age (years)	-	55.98 ± 15.30
Sex	Male	204 (66.9%)
	Female	101 (33.1%)
Insurance type		
	BPJS Kesehatan	297 (97.4%)
	Private/OOP	8 (2.6%)
Clinical outcomes		
Length of stay (days)	-	6 (1-40)*
Discharge status	Alive	295 (96.7%)
	Deceased	10 (3.3%)

Quantitative data completeness

The aggregate completeness across all 82 variables was 77.2%. There were significant disparities between domains. The highest completeness was observed in inpatient therapy (99.5%), reflecting strict adherence to e-prescribing protocols. Diagnostic data (87.4%) and Diagnosis & Classification (83.7%) also scored high. Conversely, the lowest completeness was found in Medical History (40.4%).

While major risk factors like Hypertension (98.0%) and Diabetes (96.1%) were well-documented, specific comorbidities required by international registries (e.g., thyroid disease, cancer history, sleep apnea) were rarely recorded if negative. Table 3 provides the summary of completion of each clinical domain.

**Table 3:** Aggregate completeness of core data elements by clinical domain

Domain	Number of variables	Completeness (%)
Demographics & administrative	11	81.8
Medical history	23	40.4
Clinical assessment	12	74.9
Diagnostics	12	87.4
Diagnosis & classification	4	83.7
Inpatient therapy	5	99.5
Discharge & outcomes	15	72.6
Total / Mean	82	77.2

Zero-completeness variables

Three critical variables showed 0% structured completeness in the discharge summary: New York Heart Association (NYHA) functional class (admission & discharge), discharge weight, and the National Identity Number (NIK). While vital signs like blood pressure were recorded in 100% of cases, discharge weight-crucial for monitoring fluid status-was absent from the physician's discharge summary, despite being recorded in nursing notes.

Qualitative findings

The thematic analysis of interview transcripts revealed five major themes explaining the quantitative gaps: (1) the influence of reimbursement on coding (the "BPJS-mindset"); (2) a "positive findings bias" regarding medical history; (3) technical barriers necessitating workarounds; (4) interprofessional data fragmentation; and (5) terminological mismatches regarding functional class assessment. Representative quotes and detailed descriptions for each theme are presented in Table 4.

**Table 4:** Key themes and representative quotes from informants ADHF: Acute Decompensated Heart Failure; BPJS Kesehatan: Badan Penyelenggara Jaminan Sosial Kesehatan (Indonesia's National Health Insurance); EMR: Electronic Medical Records; NYHA: New York Heart Association.

Theme	Description	Representative quotes
The "BPJS-Mindset" and administrative coding	Documentation habits and coding choices are primarily shaped by reimbursement requirements rather than clinical precision or research needs.	"Our medical records now adjust to (National Health Insurance) requirements... the mindset is already a 'BPJS-mindset'. So the resume content is tailored for BPJS to ensure the claim goes through." (Informant 01, Cardiologist) "We still use the old ICD-10 version... the application has not been updated. So, many specific ADHF diagnoses do not have codes yet, and finally, we use an approximation (I50.9)."(Informant 04, Medical Records)
Positive findings bias	Clinicians tend to omit "negative" findings (pertinent negatives) to save time, which the registry audit interprets as missing data.	"What is written is only the positive findings. Clinicians don't have enough time to write down thyroid history or cancer history if the results are negative, especially if we already know the patient well." (Informant 02, Cardiologist)
Technical workarounds and system latency	System bugs (e.g., "interconnectedness") and slow performance force residents to draft notes in external apps, preventing structured data entry.	"The application is slow... We feel like we are fighting the application. Also, if we open two medical records at once, the data can get 'interconnected' (swapped). So, we often type everything in Google Docs first, then copy-paste it to the resume, especially for diagnostics." (Informant 06, Resident)
Data fragmentation	Critical clinical data collected by nurses (e.g., daily weights) is not automatically visible or transferred to the physician's discharge summary.	"Actually, (daily weight) is in the integrated progress notes; nurses measure it daily for fluid balance. But it indeed isn't listed in the (physician's) resume because it needs to be added manually." (Informant 07, Head Nurse)
Terminological mismatch & clinical relevance	Clinicians prioritize hemodynamic profiles (Stevenson) for acute management over NYHA, or use non-standard abbreviations ("FC") that are missed by the EMR analysis.	“For inpatient care, we don't write NYHA because ADHF is automatically Class 3 or 4. We use the Stevenson classification so the therapy is clear. So (the assessment) is in the narrative, but it is not explicitly written as 'NYHA'."(Informant 01, Cardiologist) “Actually, it's just to abbreviate... So we sometimes write 'Heart Failure Functional Class’ or just 'FC' in the diagnosis sometimes, but mostly we use wet warm classification." (Informant 05, Resident)

Regarding the technical barriers (Theme 3), a follow-up discussion with the Head of the Hospital Information System (SIRS) clarified that the in-house EMR is technically capable of supporting features like dropdown menus and auto-population. This suggests the primary constraint is organizational policy and the need of an update regarding hospital data governance, rather than inherent software limitations.

## Discussion

This study represents the first comprehensive analysis of data readiness for an ADHF registry at Indonesia's National Cardiovascular Center. The findings revealed a sharp contrast: the institution excels in documenting acute procedural care (99.5% completeness in therapy), but struggles with longitudinal and preventive variables required for high-quality registries.

The "Younger, Sicker" Asian phenotype

The demographic findings (mean age ~56 years) corroborate data from the ASIAN-HF registry, which noted that Southeast Asian patients present nearly a decade younger than their Western counterparts (e.g., average age 66 in the US Change the Management of Patients with Heart Failure (CHAMP-HF) registry) [5,12]. This reinforces the urgent need for a local registry to study the etiology of premature HF in Southeast Asia, specifically Indonesia.

Coding issue and lost clinical data

While clinical narratives often contained rich detail regarding etiology (e.g., ischemic, valvular), each patients' final administrative code with ADHF was generalized to I50.9 ("heart failure, unspecified" in ICD-10). This is exacerbated by the Casemix (Indonesian Case-Based Groups or INA-CBG) system, where the use of broad diagnostic codes is favored to minimize administrative friction and the risk of dispute during the verification process. By "downcoding" patients to unspecified HF, the hospital avoids claim rejection. By not writing the actual specific ICD-10 codes, the clinical data is lost to generic coding, thus masking the true complexity of the patient population, limiting the use of administrative data for research, necessitating manual chart abstraction, which is resource-intensive [7], and limiting the ability to stratify risk associated with the diagnosis.

NYHA and the structure of clinical data

The complete absence (0%) of structured NYHA classification is a major barrier, though it does not necessarily imply a lack of clinical assessment. Qualitative interviews revealed a terminological mismatch: clinicians frequently documented "Functional Class" (FC) or narrative descriptions of effort tolerance, which were not captured by the specific "NYHA" field search. Furthermore, in acute management setting, clinicians prioritized the Stevenson profile ("Wet/Dry," "Warm/Cold") over NYHA, viewing functional class as an outpatient metric or assuming admission status to either be NYHA III-IV by default. However, without capturing a structured NYHA class specifically at discharge, the registry cannot track functional recovery or predict readmission risk effectively [13].

Patient safety implications of technical instability

A critical finding was the 'interconnectedness bug,' where data from one patient could inadvertently populate another patient's record. This presents a risk to patient safety regarding data accuracy, and if undetected, could theoretically lead to erroneous clinical decision-making. Furthermore, the workaround adopted by residents such as drafting notes in external cloud-based processors before pasting them into the EMR, introduces significant medicolegal liabilities and data privacy risks. This highlights that EMR stability is not merely a documentation convenience but a fundamental requirement for safe clinical practice.

Interoperability and the zero variables

The absence of the National Identity Number (NIK) and discharge weight in the extracted dataset highlights technical fragmentation. While the NIK is visible to clinicians in the backend, its extraction for research is currently hindered by strict interpretations of the Personal Data Protection Law (UU PDP). This restriction presents a significant paradox: the NIK is the essential Single Identity Number (SIN) required for the Ministry of Health’s SatuSehat platform to track long-term survival and readmissions across the national hospital network.

Indonesia has already successfully demonstrated the utility of NIK-based integration during the COVID-19 pandemic, where it served as the unique identifier for the PeduliLindungi ecosystem to track vaccination status and mobility nationwide [14]. To reconcile these privacy concerns with research needs, RSJPDHK should implement a pseudonymization framework. By converting the raw NIK into a cryptographic hash (a unique, irreversible code) for research datasets, the institution can enable longitudinal tracking and national linkage without exposing sensitive personal data. This approach mirrors the successful use of the Personal Identity Number (Personnummer) in the Swedish Heart Failure Registry (SwedeHF), which balances rigorous privacy protection with high-fidelity outcome tracking [15].

Furthermore, the gap between the nursing and physician’s integrated patient development report (CPPT) results in lost data (e.g., discharge weights). Finally, our interviews revealed that the hospital's in-house EMR is technically capable of bridging this gap. The barrier is not software capability but rather the need for institutional policy to mandate specific clinically relevant interface changes, such as auto-population and dropdown fields.

Limitations

This study has several limitations. First, as a single-center analysis conducted at a national tertiary referral center, the findings regarding infrastructure and staffing may not possess external validity for smaller community hospitals with different resources. Second, the analysis primarily assessed data completeness (the presence of information) rather than data accuracy. We did not validate the clinical correctness of diagnoses against a gold standard, nor did we assess patient-level outcomes such as post-discharge mortality in relation to data quality. Third, the retrospective design limits the ability to establish causal relationships between the identified systemic barriers and specific documentation behaviors. Finally, the qualitative analysis carries inherent observer bias. While we attempted to mitigate this through data triangulation across multiple professional roles and independent coding, the interpretation of themes remains subjective. These findings should be viewed as a foundational pilot for Indonesia's cardiac digital health transformation rather than a universal representation of the national healthcare landscape.

## Conclusions

The National Cardiovascular Center Harapan Kita possesses a robust administrative dataset, but it is currently insufficient for a high-quality clinical ADHF registry due to a lack of clarity in medical history, functional status, and diagnostic coding. The barriers are not merely clinical but structural: a reimbursement-driven coding culture, technical interoperability gaps, and positive findings bias in the medical history domain.

To build a world-class ADHF registry, the institution could adopt the ESC EuroHeart tiered-data model, enforcing mandatory entry for level one prognostic variables (e.g., left ventricular ejection fraction (LVEF), NYHA) while simplifying optional, additional fields for level two, and even local or regional specific fields for level three. Technical upgrades to fix the "interconnectedness bug" and auto-populate nursing data into physician summaries are immediate priorities. Finally, shifting from generic I50.9 coding to specific phenotyping will ensure the digital record accurately reflects the complex care provided, ultimately improving outcomes for the distinctively younger heart failure population in Indonesia. Furthermore, as the national cardiovascular center, RSJPDHK is positioned to lead the national cardiovascular network. The standardized data model and improved EMR architecture proposed in this study are designed not only for local use but as a scalable pilot project for other general hospitals in Indonesia. Once optimized, this registry framework is intended for implementation across the national hospital network, ensuring a sustainable, clinical outcome-driven cardiovascular data ecosystem in Indonesia.

## Appendices

Appendix 1: Semi-structured interview guide

**Table 5:** Semi-structured interview guide ADHF: Acute Decompensated Heart Failure; BPJS Kesehatan: Badan Penyelenggara Jaminan Sosial Kesehatan (Indonesia's National Health Insurance); CPPT: Catatan Perkembangan Pasien Terintegrasi (Integrated Patient Progress Notes); EMR: Electronic Medical Records; IGD: Instalasi Gawat Darurat (Emergency Department); NIK: Nomor Induk Kependudukan (National Identity Number); Poli: Polyclinic (Outpatient Clinic); UU PDP: Undang-Undang Perlindungan Data Pribadi (Personal Data Protection Law). Credit: Interview guide developed by Bun R, Adisasmito WB, Siswanto BB for this study.

Category	Question
Introduction	Could you please describe your specific role and responsibilities regarding the care of Acute Decompensated Heart Failure (ADHF) patients?
Workflow	Please describe the typical workflow for documenting a patient's journey from admission to discharge. Who is responsible for the discharge summary?
Completeness	Our analysis shows low documentation for comorbidities (e.g., thyroid, cancer). Is this due to a lack of assessment or a documentation culture?
Technical	Structured data for NYHA Class was 0%. Do you assess functional status? Why is it not explicitly written as "NYHA Class"?
	How would you describe the user experience of the current EMR system? Are there issues with speed or bugs?
	Does data flow automatically between the nursing module (CPPT) and the physician module (Resume)?
Coding	Why is the code I50.9 (Heart Failure, Unspecified) used almost exclusively? Is this driven by clinical uncertainty or reimbursement?
Closing	If you could change one thing about the current EMR system to make data entry easier, what would it be?
